# The influence of the vocal tract on the attack transients in clarinet playing

**DOI:** 10.1080/09298215.2019.1708412

**Published:** 2020-01-20

**Authors:** Montserrat Pàmies-Vilà, Alex Hofmann, Vasileios Chatziioannou

**Affiliations:** Department of Music Acoustics, University of Music and Performing Arts Vienna, Vienna, Austria

**Keywords:** Clarinet, transients, vocal tract, performance analysis, music acoustics

## Abstract

When playing single-reed woodwind instruments, players can modulate the spectral content of the airflow in their vocal tract, upstream of the vibrating reed. In an empirical study with professional clarinettists ($N_{p}=11$), blowing pressure and mouthpiece pressure were measured during the performance of Clarinet Concerto excerpts. By comparing mouth pressure and mouthpiece pressure signals in the time domain, a method to detect instances of vocal tract adjustments was established. Results showed that players tuned their vocal tract in both clarion and altissimo registers. Furthermore, the analysis revealed that vocal tract adjustments support shorter attack transients and help to avoid lower bore resonances.

## 1. Introduction

In single-reed woodwind instruments, the oscillations of a single reed clamped to a mouthpiece are responsible for the sound generation. The reed-mouthpiece system (non-linear excitation mechanism) is acoustically coupled to the resonance of the instrument bore (linear resonator) (Nederveen, 1969). Globally, the system is controlled by the actions of the player. Traditionally in music acoustics, the actions of the player have been considered as mainly controlling blowing pressure, embouchure and fingerings, thus defining the loudness, the timbre and the frequency of the sound. Over the last decades, the influence of the player's vocal tract on the sound production has gained larger research interest (e.g. Chen, Smith, & Wolfe, 2011; Scavone, Lefebvre, & da Silva, 2008).

**Figure 1.**
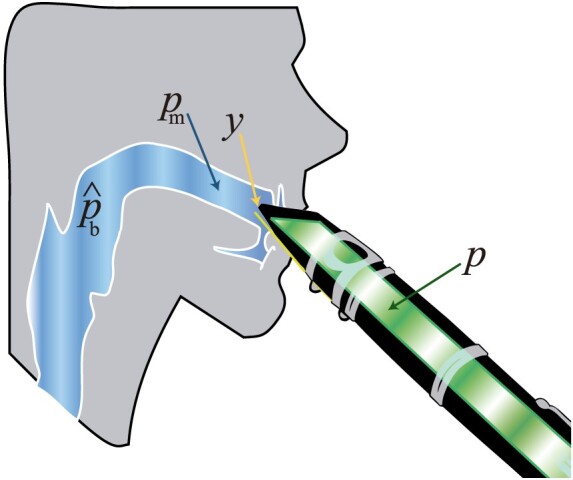
Schematic of the *mouth-reed-mouthpiece system* showing blowing pressure (${\hat{p}}_{b}$) and dynamic mouth pressure ($p_{m}$) in the upstream resonator (blue), mouthpiece pressure (*p*) in the downstream resonator (green) and reed-tip displacement (*y*).

The vocal tract is the space in the player's respiratory system between the larynx and the lips, in which the air column can resonate upstream of the reed. The shape of the vocal tract is responsible for producing different vowels in speech (Stevens & House, 1961). In a similar way, clarinet players claim to use different vowel-like shapes in the vocal tract during playing (González & Payri, 2017). Considering the vocal tract as an additional resonator, the reed of a woodwind instrument can be seen as an oscillating valve acting between two cavities: the bore of the instrument (downstream resonator) and the vocal tract of the player (upstream resonator), as schematised in Figure 1. Both cavities can resonate at several frequencies depending on the length and geometry of the bore of the instrument (Nederveen, 1969), and the shape of the player's vocal tract (Wilson, 1996).

**Figure 2.**
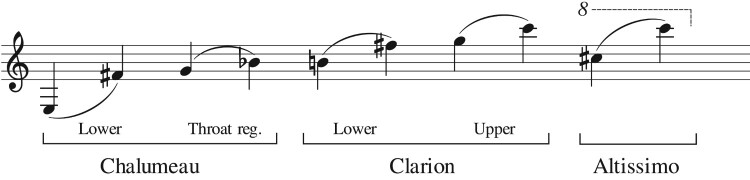
Pitch range of the registers in the B♭ clarinet.

The B♭ clarinet is divided into three registers: the low or chalumeau (written E3 to B♭4; sounding 147–415 Hz), the medium or clarion (written B4–C6; sounding 440–932 Hz) and the high or altissimo register (from written C♯6, sounding 988–1760 Hz approx.), as shown in Figure 2. In the chalumeau register, the clarinet plays at the fundamental frequency corresponding to the tube length, while the upper registers make use of a register key. In the chalumeau register, the upper tones (G to B♭) are achieved by opening side holes and are called ‘throat register’ as the player might modify the oral settings to improve the sound quality (Wilson, 1996). In the clarion register, González and Payri (2017) proposed to differentiate the lower and the upper part, as indicated in Figure 2, as they might require different conscious vocal tract adjustments.

The first arguments supporting the influence of the vocal tract on the sound production on single-reed woodwinds were raised by Clinch, Troup, and Harris (1982) and Benade and Hockje (1982). Clinch et al. (1982) highlighted that the vocal tract influence depends on the register of the instrument, and Benade and Hockje (1982) pointed out that the role of the vocal tract might be crucial for the tone onsets. Backus (1985, p. 17), however, concluded that ‘the vocal tract has a negligible influence on the instrument tone’, justifying why most models of single-reed woodwind instruments do not consider the vocal tract. Nevertheless, subsequent studies showed that the adjustments of the vocal tract are used by players to perform special musical effects, like glissandi or pitch bending, and allow to play tones above the natural range of the instrument (Wilson, 1996).

A method to assess the influence of the vocal tract during playing was presented by Wilson (1996), considering the Elliott and Bowsher (1982) formulation. Defining the impedance of a resonator as z=p/u, where *p* is the pressure and *u* is the volume flow in the steady state, and assuming flow conservation between the two cavities, one can write:

(1) $$\frac{p_{\mathrm{mouthpiece}}}{p_{\mathrm{mouth}}}=\frac{z_{\mathrm{mouthpiece}}}{-z_{\mathrm{mouth}}}.$$

Strictly, this equation cannot be applied during the transient. However, this formulation allows to indirectly obtain the mouth-to-mouthpiece impedance ratio by considering the mouth-to-mouthpiece pressure ratio. Following this concept, Scavone et al. (2008) demonstrated the influence of the vocal tract on saxophone performance, by analysing the pressure in the instrument bore and in the player's mouth in the frequency domain. A similar analysis was used on an instrumented saxophone (Guillemain, Vergez, Ferrand, & Farcy, 2010) and on a trombone (Fréour & Scavone, 2013). Both Scavone et al. (2008) and Guillemain et al. (2010) showed the role of the vocal tract when playing glissandi, bugling or playing multiphonics.

Using a different experimental methodology, Chen, Smith, and Wolfe (2009) analysed the effect of the vocal tract on the clarinet and on the saxophone (Chen et al., 2011; Li, Chen, Smith, & Wolfe, 2015) via impedance measurements of the oral cavity during playing. With an analysis in the frequency domain, they demonstrated that the peaks of the impedance curve of the upstream resonator correlate with the played frequency and concluded that players configure their vocal tract in order to select certain harmonics. Similar observations were made by Lulich, Charles, and Lulich (2017) using ultrasound imaging (frame rate smaller than 20 fps) to analyse the tongue shape during clarinet playing. Although Benade and Hockje (1982) and later Guillemain (2007) emphasised the importance of the vocal tract during attack transients, most studies on the vocal tract were confined to the steady-state part of the tones.

Transient phenomena occur at the beginning and at the end of tones, when the amplitude of the oscillations varies. In single-reed woodwind instruments, transient phenomena have been extensively analysed both theoretically and experimentally (Almeida, Li, Smith, & Wolfe, 2017; Chatziioannou, Schmutzhard, Pàmies-Vilà, & Hofmann, 2019; Li, Almeida, Smith, & Wolfe, 2016; Pàmies-Vilà, Hofmann, & Chatziioannou, 2018). It has been found that attack and release transients play an important role on the timbre perception of instruments (Fletcher & Rossing, 1998), and their analysis contributes to the understanding of sound generation (Almeida et al., 2017). Since the two aforementioned methods to observe vocal tract modifications (pressure comparison or impedance measurements) are performed in the frequency domain, they provide few insight into transient phenomena. In order to observe the transients occurring at the tone attack, release and during the note transitions, the current analysis is performed in the time domain. In a previous study, Pàmies-Vilà, Scavone, Hofmann, and Chatziioannou (2017) presented a time-domain analysis to detect instances of vocal tract adjustment in saxophone playing. This method was used to show vocal tract effects when playing extended techniques (pitch bending and overtones) and also during note transitions in legato playing.

The current study aims at detecting instances of vocal tract adjustment during clarinet performance in order to analyse the vocal tract influence across different registers. Section 2 presents the experimental approach and the methodology to analyse the data obtained with 11 clarinettists performing excerpts of a Clarinet Concerto. Sections 3 and 4 expose and discuss the results of this analysis with a special focus on vocal tract effects on transient phenomena.

## 2. Materials and methods

### 2.1. Experimental setup

**Figure 3.**
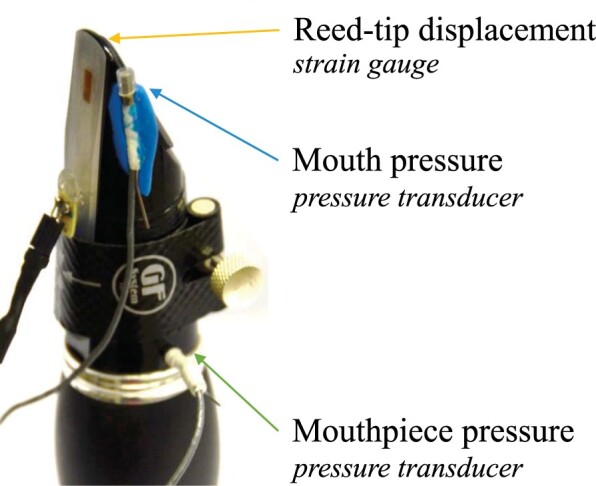
The pressure transducers and strain-gauge reed attached to a German B♭-clarinet mouthpiece for vocal tract measurements.

For this study, a German B♭ clarinet and mouthpiece (Thomann GCL-416 Synthetic Line; Maxton NA-1) are equipped with sensors (Figure 3). The mouth pressure and the mouthpiece pressure are measured with two pressure transducers (Endevco 8507C-2). The first transducer is attached on the side of the mouthpiece, so that it reaches inside the player's mouth. The second transducer is inserted into the mouthpiece via an orifice at 7.5 cm from the mouthpiece tip. The pressure signals are conditioned using the corresponding amplifier (Endevco model 136).

In order to measure the reed-tip displacement and to trace the tongue–reed interaction, the reed vibration is measured with a strain gauge (length of 2 mm) attached to the surface of a synthetic reed (strength 2.5, German cut, by Légère), as used in Hofmann and Goebl (2016). The external sound is recorded under dry acoustic conditions, with a microphone (d:vote 4099U, by DPA) mounted on the instrument. All signals are simultaneously recorded at a sampling rate of 50 kHz using a digital acquisition platform (NI 9220, by National Instruments). The recorded data can be found online at https://doi.org/10.21939/tpisrwi.

### 2.2. Participants and experimental procedure

**Figure 4.**
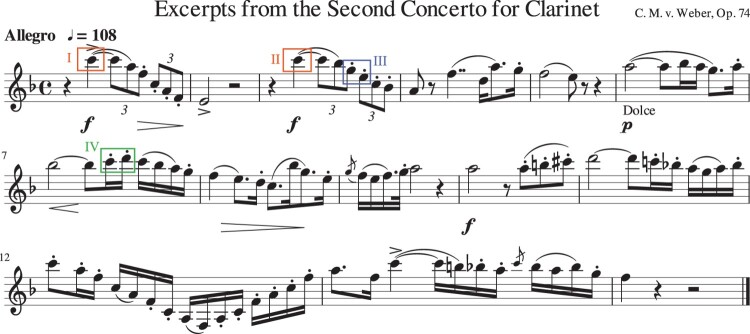
One of the excerpts of the Clarinet Concerto n. 2 in E♭ Major op. 74 by Carl Maria von Weber. Notes I–IV are analysed in Figures 5, 8 and 9.

Eleven clarinet players ($N_{p}=11$) were invited to play articulation exercises and a selected piece of music. Among the participants there were nine advanced students and two professional players. All players were over 18 years old (average of 23 years old) and had clarinet playing as their profession, with 7–20 years of experience. The results regarding the articulation exercises were presented in Pàmies-Vilà et al. (2018). An analysis of the data captured with the musical piece is presented in this article. In order to analyse realistic playing conditions covering a wide pitch range of the instrument, three musical excerpts were selected from the Clarinet Concerto No. 2 in E♭ Major op. 74 by Carl Maria von Weber (see first excerpt in Figure 4). This piece was known by all players, as it is part of the repertoire used in the admission examination to the University.

**Figure 5.**
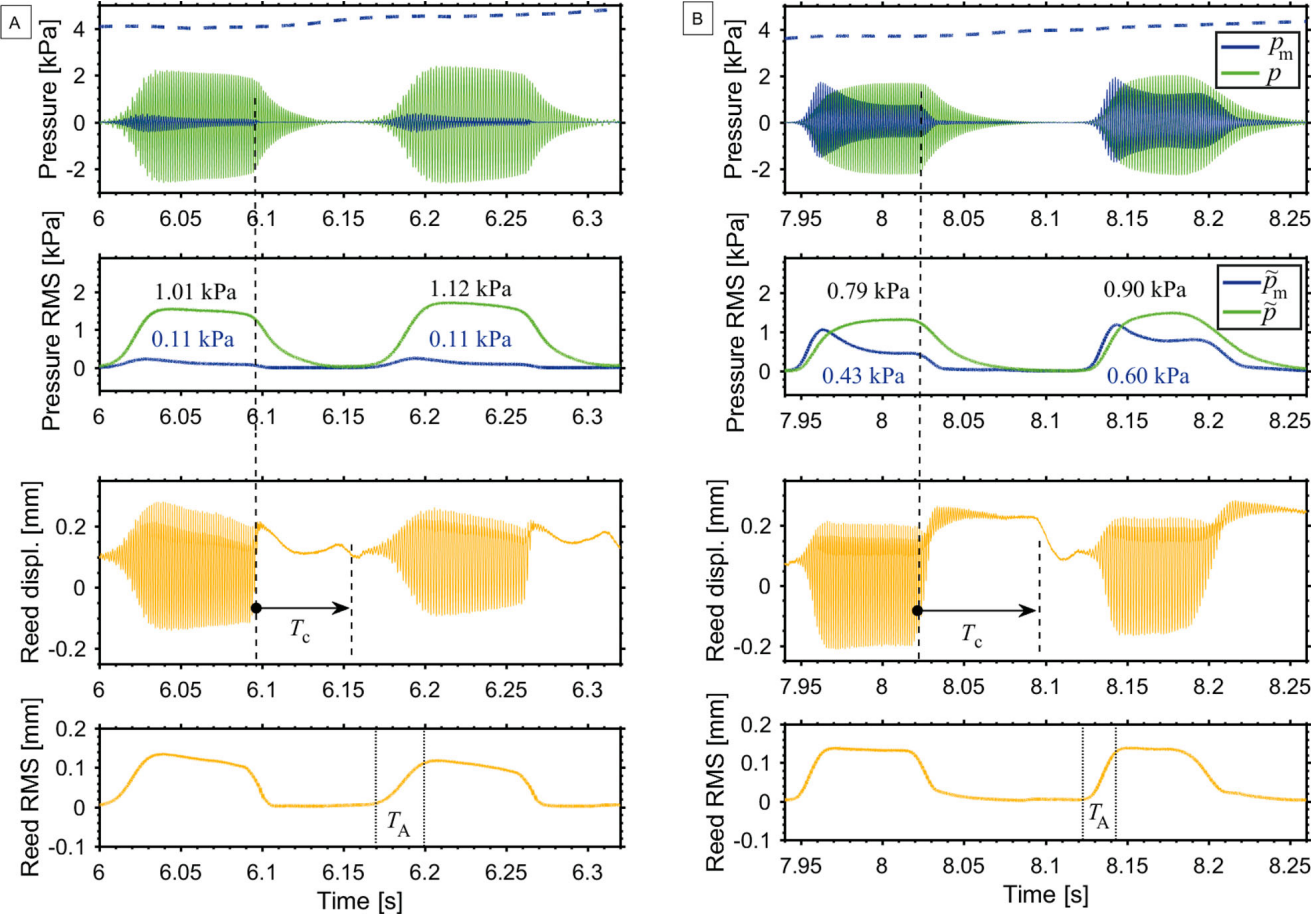
Two tones played in staccato articulation (written G5 and E5; box III in Figure 4), without (A) and with (B) vocal tract adjustment. The top plots show the blowing pressure (dashed line), mouthpiece pressure *p*, and dynamic mouth pressure $p_{m}$ signals, with their corresponding RMS envelopes ($\tilde{p}$ and ${\tilde{p}}_{m}$), and an indication of the RMS values per note. On the reed-tip displacement, $T_{c}$ shows the tongue–reed contact duration. The bottom plots show the RMS envelope of the high-pass-filtered reed-tip displacement; $T_{A}$ indicates the attack-transient duration. [Players n. 1 and n. 6]

**Figure 6.**
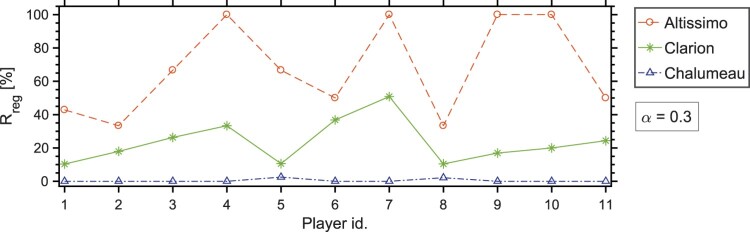
Percentage of tones played with vocal tract adjustment ($p_{m,\mathrm{RMS}}>\alpha p_{\mathrm{RMS}}$, with α=0.3), calculated as in Equation (3), for the different registers of the B♭-clarinet (altissimo, clarion, chalumeau) for all 11 players.

**Figure 7.**
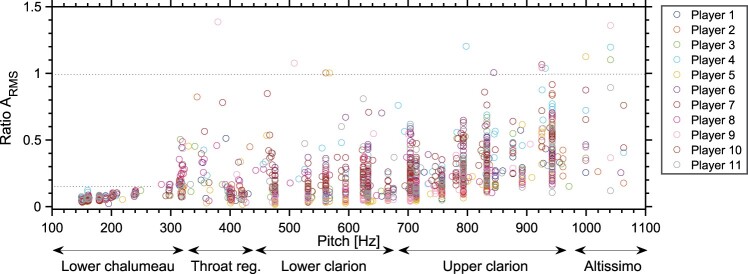
Ratio between the RMS values of the mouth and mouthpiece pressures $A_{\mathrm{RMS}}=p_{m,\mathrm{RMS}}/p_{\mathrm{RMS}}$ as a function of pitch, for all tones and players.

**Figure 8.**
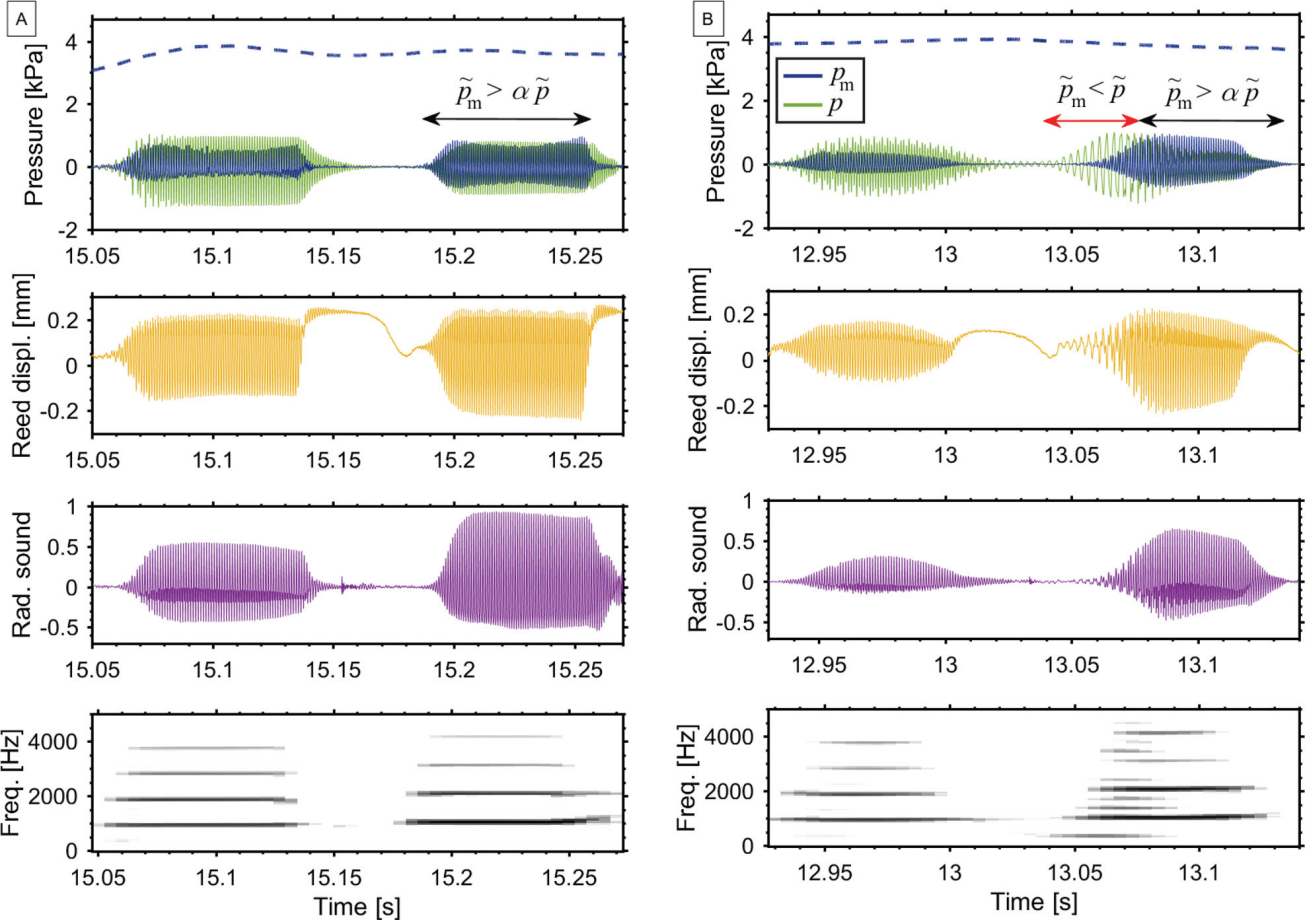
Two tones played in staccato articulation (written C6 and D6; box IV in Figure 4) by two clarinettists, A and B. The top plots show blowing pressure (dashed line), mouthpiece pressure *p*, dynamic mouth pressure $p_{m}$, and reed-tip displacement. The bottom plots show the normalised radiated sound and its spectrogram. [Players n. 11 and n. 2]

**Figure 9.**
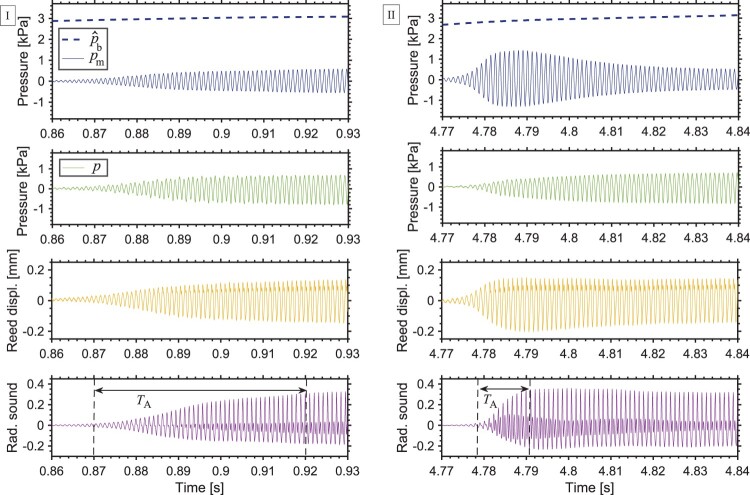
Attack transient measured for a written C6 (highest tone of the clarion register) at (I) the beginning of the music excerpt and (II) when repeating the same pattern (boxes (I) and (II) in Figure 4) as played by one player. Plots show blowing pressure ${\hat{p}}_{b}$ (dashed line) and dynamic mouth pressure $p_{m}$, mouthpiece pressure *p*, reed-tip displacement and normalised radiated sound. $T_{A}$ shows the duration of the attack transient. [Player n. 4]

Following the experimental protocol approved by the Ethics Committee of the University of Music and Performing Arts Vienna, the participants were informed about the experimental procedure, agreed to an anonymous usage of their data and gave written consent of their voluntary participation in the experiment, for which they received a nominal fee.

All participants performed on the same sensor-equipped clarinet. Before the actual recording, each participant had about 10 min of warm-up time to get used to the instrument. Every session lasted for about 40 min to 1 h. At the end of the session, the participants filled a form with information about their playing technique and music practice.

### 2.3. Data processing

#### 2.3.1. Signal conditioning

The signals captured in this experiment are the acoustic pressure in the mouth, the acoustic pressure in the mouthpiece, the reed-surface strain and the radiated sound. The pressure measured in the mouth consists of both a static (DC) and a dynamic (AC) component. The static component corresponds to the pressure used to blow into the instrument (hereafter blowing pressure ${\hat{p}}_{b}$). The dynamic component expresses the sound vibration, which shows the resonance of the upstream resonator (hereafter dynamic mouth pressure $p_{m}$). The total pressure measured in the player's mouth equals ${\hat{p}}_{b}+p_{m}$. As the instrument bore (downstream resonator) is open to the atmospheric pressure, the mouthpiece pressure *p* consists only of a dynamic component (variables indicated in Figure 1).

The blowing pressure ${\hat{p}}_{b}$ (DC component, dashed signal around 4 kPa in Figure 5) is obtained after low-pass filtering the measured mouth pressure using a moving-average filter. The filter is applied with time windows of length $T_{L}=6.8\;\mathrm{ms}$, with $T_{L}$ being the length of one period of the lowest played tone. Then, the dynamic mouth pressure $p_{m}$ (AC component) is obtained by subtracting the blowing pressure ${\hat{p}}_{b}$ from the measured signal: $p_{m}=p_{\mathrm{measured}}-{\hat{p}}_{b}$. This pressure signal $p_{m}$ is hence centred at 0, allowing comparison with the mouthpiece pressure *p*, as shown at the top of Figure 5. The strain-gauge signal is calibrated to obtain the reed-tip displacement *y*, as detailed in Pàmies-Vilà, Hofmann, and Chatziioannou (2017).

#### 2.3.2. Attack transients detection

The attack transient refers to the transitory phase at the tone onset when the amplitude of oscillation rises. As shown in Figure 5(B), the attack transients do not appear simultaneously in the mouthpiece pressure and in the dynamic mouth pressure. For this reason, the reed-tip displacement signal *y* is used to determine the location and duration of every attack transient. This signal is first high-pass filtered, by subtracting its DC component (computed with a moving-average filter, with time windows of length $T_{L}=6.8\;\mathrm{ms}$, with $T_{L}=6.8\;\mathrm{ms}$ being the length of one period of the lowest played tone). Then, the root-mean-square envelope $\tilde{y}$ of the signal is computed, using time windows of length $1.5\;T_{L}$. The attack transients correspond to the rising part of the envelope $\tilde{y}$ at every tone onset, as indicated with $T_{A}$ in the bottom of Figure 5. The location of the attack transients is detected when the first derivative of $\tilde{y}$ is positive. The duration of the attack and release transient is computed between the instances where the envelope is at 5% and 95% of the amplitude in the steady state. Once all the attack transients have been detected in the reed signal, the recorded signals are split into tones, from the beginning of an attack transient, to the beginning of the next one.

#### 2.3.3. Detecting vocal tract adjustments

Two different performances are compared in Figure 5, as played by two participants A and B. The tones are extracted from a passage of the Clarinet Concerto that is played in staccato articulation in the clarion register (written G5 and E5; box III in Figure 4). The mouthpiece pressure *p* overlaps the dynamic mouth pressure $p_{m}$ for comparison. At the top, the blowing pressure (dashed signal) remains almost constant around 4 kPa. The tones are articulated by stopping the reed vibration with a tongue contact. On the reed-tip displacement, the vertical dashed lines show the tongue–reed contact duration, indicated as $T_{c}$.

An example when playing without vocal tract adjustment is shown in Figure 5(A). Here the amplitude of the dynamic mouth pressure $p_{m}$ is more than 5 times smaller than the amplitude of the mouthpiece pressure *p*. In contrast, in Figure 5(B) the amplitude of the dynamic mouth pressure $p_{m}$ is comparable to the amplitude of the mouthpiece pressure *p*, which is unlikely to occur without voluntary action of the player. It is worth noticing that, in both A and B examples, the amplitude of the dynamic mouth pressure rises faster than that of the mouthpiece pressure. A reason may be the wider impedance peaks of the mouth-resonator (lower quality factor) compared to the instrument-resonator, which results in shorter attack and release transients in the dynamic mouth pressure (Guillemain et al., 2010). Similarly, after the instant of tongue–reed contact (vertical dashed line), the dynamic mouth pressure $p_{m}$ decays faster than the mouthpiece pressure *p*, because the vocal tract dampens the sound vibration faster than the instrument bore.

In order to compare the amplitudes of the oscillations of $p_{m}$ and *p*, the Root-Mean-Square (RMS) level is calculated for both pressure signals at every tone. The RMS level provides a measure of the energy of the signal at the considered time window. For the discrete points n=1,…,N of a signal *x*, the RMS level is calculated as

(2) $$x_{\mathrm{RMS}}=\sqrt{\frac{1}{N}\sum_{n=1}^{N}|x_{n}{|}^{2}}.$$

After having split the recorded signals into single tones, the RMS level is computed for every tone for the mouthpiece pressure $p_{\mathrm{RMS}}$ and for the dynamic mouth pressure $p_{m,\mathrm{RMS}}$, respectively. Doing so, one can compare the amplitude of the mouth and mouthpiece oscillations with a single value per tone, giving a measure of the vocal tract influence relative to the amplitude of the sound in the mouthpiece. The root-mean-square values of the tones in Figure 5 are given together with the RMS envelopes of $p_{m}$ and *p* for comparison. These RMS envelopes are computed using time windows of length $1.5\;T_{L}$ (with $T_{L}=6.8\;\mathrm{ms}$ being the length of one period of the lowest played tone), and indicated as ${\tilde{p}}_{m}$ and $\tilde{p}$, respectively.

#### 2.3.4. Pitch detection and register

The vocal tract influence on the sound production in single-reed woodwinds changes with register and it is more prominent for high tones (Clinch et al., 1982; Scavone et al., 2008). Towards a pitch detection algorithm, the mouthpiece pressure signal is split into windows of length $3\;T_{L}$ (with $T_{L}=6.8\;\mathrm{ms}$ being the period of the lowest played tone), with a 50% overlap. Auto-correlation within each window is computed to obtain the period of the oscillation following the algorithm by McLeod and Wyvill (2005). This algorithm has a time (approx. 5 ms) and frequency resolution that allows to follow pitch changes during transients. The detected frequencies are grouped by register as: chalumeau (below 430 Hz), clarion (between 430 and 965 Hz) and altissimo (above 965 Hz), according to Figure 2.

## 3. Results

### 3.1. Vocal tract adjustments for different registers

In order to assess how often a player shows vocal tract adjustments, a reference value is computed for every register. The condition $p_{m,\mathrm{RMS}}>\alpha p_{\mathrm{RMS}}$, with *α* being a positive number in (0,1), is used to compare the RMS values of the dynamic mouth pressure and mouthpiece pressure. The reference value is obtained for each register as the ratio of the number of tones with vocal tract adjustment $n_{\mathrm{reg}}$ over the total number of tones detected in every register $N_{\mathrm{reg}}$, namely

(3) $$R_{\mathrm{reg}}=\frac{\left\{n_{\mathrm{reg}}\;|\;p_{m,\mathrm{RMS}}>\alpha p_{\mathrm{RMS}}\right\}}{N_{\mathrm{reg}}}.$$

The values of $R_{\mathrm{reg}}$ for every player and register are plotted in Figure 6, with α=0.3. This ratio expresses how often the tones are played with vocal tract adjustments. The results show significant differences among registers and among players. In addition to previous observations (Chen et al., 2009; Wilson, 1996), players do not only show wider mouth-pressure amplitudes when playing particular musical effects or extended register, but also during ordinary playing (e.g. playing a classical Clarinet Concerto).

Regarding the register (see registers in Figure 2), Figure 6 shows that both the altissimo and the clarion register present tones where the dynamic mouth pressure is comparable to the mouthpiece pressure, but almost no such instances are detected in the chalumeau register. Furthermore, players show vocal tract adjustments more often in the altissimo than in the clarion register. These observations are in agreement with a number of previous studies, which associate vocal tract effects with playing high tones or overtones (Chen et al., 2009; Clinch et al., 1982; Scavone et al., 2008).

Regarding individual playing characteristics, some participants show vocal tract adjustments more often (players 4, 6, 7 in the clarion; players 4, 7, 9 and 10 in the altissimo in Figure 6) than other participants (players 1, 2, 5, 8). The clarion register presents less variability among players (vocal tract adjustment is detected at 10–40% of the played tones) than the altissimo register, which is more player-dependent. These differences suggest that the observed effects are not only register-dependent but also player-dependent, thus indicating the importance of the player's intention in the vocal tract adjustment. For every participant, we took notice of the years of practice, and the hours of practice per week, but no correlation is found between these two parameters and the results of the experiment. In addition, no significant correlation is found when considering gender or age.

Considering all tones played during the experiment, the ratio between the RMS level of the dynamic mouth pressure and the RMS level of the mouthpiece pressure is computed per note as $A_{\mathrm{RMS}}=p_{m,\mathrm{RMS}}/p_{\mathrm{RMS}}$. In Figure 7, this ratio $A_{\mathrm{RMS}}$ is plotted as a function of pitch, with indication of the sub-registers as in Figure 2. The higher values of $A_{\mathrm{RMS}}$ indicate that $p_{m,\mathrm{RMS}}\approx p_{\mathrm{RMS}}$, i.e. the vocal tract adjustment is considered significant.

In the lower chalumeau register, $A_{\mathrm{RMS}}$ is below 0.15 (Figure 7), showing that the dynamic mouth pressure oscillates at significantly lower amplitudes than the mouthpiece pressure, hence no vocal tract adjustment is detected. In the throat register and in the lower clarion register some values of $A_{\mathrm{RMS}}$ are close to 1.0, as in some cases the vocal tract adjustment becomes significant. In the upper clarion register, the values of $A_{\mathrm{RMS}}$ increase with pitch, showing the highest $A_{\mathrm{RMS}}$ between 800 and 950 Hz, where all players show vocal tract adjustments. In the upper-clarion and altissimo registers, where many players show high values of $A_{\mathrm{RMS}}$, particular effects have been observed, as it is next explored in Section 3.2.

### 3.2. Vocal tract adjustments during the production of high tones

For high tones (upper clarion and altissimo registers), the analysis shows that vocal tract adjustments are required in order to assure precise attack transients and to obtain the targeted pitch. Poorly performed high-pitch tones often result in a squeak—a pitch higher than the targeted one—or in an undertone—when a tone at a lower resonance is played instead of the targeted pitch, also called stray noise (Benade, 1985). When playing a bugling exercise (playing tones from the harmonic series while maintaining a certain fingering), the vocal tract can control which harmonic is played (Scavone et al., 2008). A similar practice is found in classical playing, to avoid the appearance of other tones that are harmonically related to the fingered tone.

Measurements considering two different participants, A and B, are shown in Figure 8. The signals show the same two tones in a fast passage with staccato articulation (box IV in Figure 4): a written C6 (highest tone of the clarion register) followed by a D6 (altissimo register). Both players show a significantly high dynamic mouth pressure in the first tone, which allows them to achieve the C6 pitch. However, for the second tone only player A obtains the targeted frequency during the entire tone, which corresponds to $p_{m,\mathrm{RMS}}>\alpha p_{\mathrm{RMS}}$. Player B fails at over-blowing into the altissimo register and the clarinet sounds at a lower resonance (350 Hz), at a 12^th^ interval below the fingered tone, as shown in the spectrogram (bottom of Figure 8(B), at t=13.05s). To correct the pitch, player B adjusts the vocal tract at t=13.07s and achieves the targeted pitch (D6) at the second part of the tone. Such a fast reaction is an indicator of the auditory–motor interactions that support immediate corrections during the performance (Zatorre, Chen, & Penhune, 2007).

Regarding differences among participants, players 1, 2, 5, 7, 8 and 10 could not achieve the D6 pitch (second tone) at this passage, performing similar to Figure 8(B). In this case $p_{m,\mathrm{RMS}}<\alpha p_{\mathrm{RMS}}$. On the other hand, for players 3, 4, 6, 9 and 11 the condition $p_{m,\mathrm{RMS}}>\alpha p_{\mathrm{RMS}}$ is matched. These players satisfactorily achieve the targeted pitch (D6). Hence, it can be deduced that appropriate vocal tract adjustments reduce the appearance of undesired lower resonances at the attack transient in clarinet performance.

Another particularity of high tones was observed when the same high pitch is played again in a similar pattern: many players changed the sound quality for the repeated tones by adjusting their vocal tract. Two high tones (C6) played by the same participant at different times are compared in Figure 9(I) and (II). The two tones are 2 bars apart (boxes (I) and (II) in Figure 4) and are part of a similar pattern in the music (high tone followed by a descending arpeggio). Eventhough both tones are achieved at the targeted frequency, and no correction in pitch is needed, tone II shows larger dynamic mouth pressure amplitude. As a result, the attack transient in the radiated sound is shorter in tone II ($T_{A}=0.012\;s$) than in tone I ($T_{A}=0.05\;s$).

Considering the mouthpiece pressure *p* in Figure 9, there is no noticeable difference in the amplitude *p* between tones (I) and (II). Also the blowing pressure ${\hat{p}}_{b}$ (dashed line) shows similar values between (I) and (II). This implies that the observed changes are not a result of the blowing action or a result of the bore acoustics. It is therefore shown that the vocal tract adjustment is responsible for the shorter attack transient: increasing the dynamic mouth pressure results in a steeper rise in amplitude of the reed-tip displacement, causing a shorter attack in the radiated sound. Considering all participants in this two-tone comparison, 7 out of 11 players show stronger vocal tract influence in tone II than in tone I, i.e. they adjust the vocal tract to affect the attack transient of the tone in the later appearance of a similar pattern. Note that this could be explained because the players might aim at a different musical expression when it comes to repeating a pattern.

## 4. Discussion

This study shows that the adjustment of the vocal tract allows to produce precise attack transients avoiding undesired stray noise. Vocal tract adjustments are assessed by comparing the energy of the oscillations in the mouthpiece and in the mouth of the player at every note. The fact that the vocal tract influence depends on pitch or register is well known (Chen et al., 2009; Clinch et al., 1982; Li et al., 2015; Scavone et al., 2008). In addition to previous observations, the current study shows that vocal tract adjustments can appear in a large part of the clarinet pitch range, including the clarion register. As was suggested by Benade (1985), the vocal tract plays an important role when playing in the altissimo register. In this register, players use vocal tract adjustments to prevent the appearance of lower bore resonances at the attack transients.

In Pàmies-Vilà et al. (2018), the authors analysed blowing and tonguing actions and their relationship to attack and release transients. Including the findings on the vocal tract influence on attack transients that are reported in the current study, a broader picture on articulation techniques can be established. On the one hand, the release transients depend on the articulation style (legato, portato, staccato) and on the technique used to stop the sound (decreasing the mouth pressure or with a tongue stop), but they do not appear to be significantly influenced by the vocal tract. On the other hand, the duration of the attack transients is influenced by both the blowing action and, as shown in this study, by the vocal tract adjustment, particularly at the upper clarion and the altissimo registers.

The presented time-domain method to investigate vocal tract adjustments during clarinet playing can be understood as an extension to the existing frequency-domain techniques. In those techniques, pressure and/or impedance measurements allow to identify high-amplitude regions in the vocal tract impedance, which are related to the vocal tract geometry. The possibility to detect short instances of vocal tract adjustment allows to combine vocal tract analysis with the study of transient phenomena. A limitation of the techniques based on pressure measurements compared to the techniques based on active impedance measurements is that the detection of vocal tract adjustments is only possible in the time intervals when a sound is produced. Hence, with the proposed method, the adjustments related to the preparation of upcoming tones during rests cannot be assessed. Furthermore, no information about the mouth resonance frequency and bandwidth is obtained when using a time-domain approach.

To sum up, with the current methodology it is possible to detect instances of vocal tract adjustment during clarinet playing. The analysis shows that players adjust the vocal tract not only for extended playing techniques or extended registers, but also for ordinary playing. Vocal tract adjustments are detectable already at the upper chalumeau tones, and are widely used in the clarion register. In the altissimo register, only the players that have fine-motor control over their vocal tract settings are able to produce undistorted attack transients. This shows that to attain the full range of the instrument, it is indispensable to adjust the vocal tract in order to avoid unintended bore resonances. The presented method can be applied to study intonation issues in other wind instruments. Wind instrument players could benefit from such a sensor-equipped instrument while practising, by receiving feedback regarding their vocal tract technique. Such equipment and feedback-tools could be used in the future to diversify the teaching-learning process and facilitate social-network-based gamified learning strategies.
